# ^18^F FDG PET/MRI with hepatocyte-specific contrast agent for M staging of rectal cancer: a primary economic evaluation

**DOI:** 10.1007/s00259-021-05193-7

**Published:** 2021-03-09

**Authors:** Felix G. Gassert, Johannes Rübenthaler, Clemens C. Cyran, Johann S. Rink, Vincent Schwarze, Johanna Luitjens, Florian T. Gassert, Marcus R. Makowski, Stefan O. Schoenberg, Marius E. Mayerhoefer, Dietmar Tamandl, Matthias F. Froelich

**Affiliations:** 1 Department of Diagnostic and Interventional Radiology, Klinikum rechts der Isar, Technical University of Munich, Ismaninger Str. 22, 81675 Munich, Germany; 2 Department of Radiology, University Hospital, LMU Munich, Marchioninistr 15, 81377 Munich, Germany; 3 Department of Radiology and Nuclear Medicine, University Medical Center Mannheim, Theodor-Kutzer-Ufer 1-3, 68167 Mannheim, Germany; 4 Department of Radiology, Memorial Sloan Kettering Cancer Center New York, New York City, NY USA; 5 Department of Biomedical Imaging and Image-Guided Therapy, Division of General and Pediatric Radiology, Medical University of Vienna, Waehringer Guertel 18-20, 1090 Vienna, Austria

**Keywords:** Cost-effectiveness, Rectal cancer, PET/MRI, Staging

## Abstract

**Purpose:**

Rectal cancer is one of the most frequent causes of cancer-related morbidity and mortality in the world. Correct identification of the TNM state in primary staging of rectal cancer has critical implications on patient management. Initial evaluations revealed a high sensitivity and specificity for whole-body PET/MRI in the detection of metastases allowing for metastasis-directed therapy regimens. Nevertheless, its cost-effectiveness compared with that of standard-of-care imaging (SCI) using pelvic MRI + chest and abdominopelvic CT is yet to be investigated. Therefore, the aim of this study was to analyze the cost-effectiveness of whole-body ^18^F FDG PET/MRI as an alternative imaging method to standard diagnostic workup for initial staging of rectal cancer.

**Methods:**

For estimation of quality-adjusted life years (QALYs) and lifetime costs of diagnostic modalities, a decision model including whole-body ^18^F FDG PET/MRI with a hepatocyte-specific contrast agent and pelvic MRI + chest and abdominopelvic CT was created based on Markov simulations. For obtaining model input parameters, review of recent literature was performed. Willingness to pay (WTP) was set to $100,000/QALY. Deterministic sensitivity analysis of diagnostic parameters and costs was applied, and probabilistic sensitivity was determined using Monte Carlo modeling.

**Results:**

In the base-case scenario, the strategy whole-body ^18^F FDG PET/MRI resulted in total costs of $52,186 whereas total costs of SCI were at $51,672. Whole-body ^18^F FDG PET/MRI resulted in an expected effectiveness of 3.542 QALYs versus 3.535 QALYs for SCI. This resulted in an incremental cost-effectiveness ratio of $70,291 per QALY for PET/MRI. Thus, from an economic point of view, whole-body ^18^F FDG PET/MRI was identified as an adequate diagnostic alternative to SCI with high robustness of results to variation of input parameters.

**Conclusion:**

Based on the results of the analysis, use of whole-body ^18^F FDG PET/MRI was identified as a feasible diagnostic strategy for initial staging of rectal cancer from a cost-effectiveness perspective.

## Introduction

Cancer is one of the most important causes of morbidity and mortality in the world, with rectal cancer being within the top 3 most cancers especially in developed countries [1].

Current therapeutic standards include a wide range of chemo- and radiotherapy, surgery, and local ablative therapies with several therapeutic options even in metastasized disease [2, 3].

Besides adequate diagnosis of local tumor extent, early detection of metastases is important as metastasis-directed therapy regimens including ablation or resection of metastases can be efficient in increasing patient overall survival [3–5]. The current diagnostic standard involves magnetic resonance imaging (MRI) of the pelvis and computed tomography (CT) scan of the chest, abdomen, and pelvis, showing relatively high sensitivity but often requiring additional workup for accurate identification and characterization of, e.g., hepatic lesions [6]. In various malignant diseases, positron emission tomography (PET)/MRI provided not only high sensitivity but also high specificity for detection of metastases, avoiding the need for additional diagnostic procedures [7, 8].

Additionally, in a study recently published by Mayerhoefer et al., PET/MRI with various clinical tracers was shown to have only slightly higher overall costs when compared with PET/CT in a range of cancer entities [9]. Compared with PET/CT, the clinical use of PET/MRI still is very limited; nevertheless, the number of PET/MRI systems worldwide is gradually increasing [10]. Furthermore, the increasing number of PET/MRI installations and possible implementation of PET/MRI in clinical practice bear the potential of lowering the cost per examination.

In a pilot study recently published, Yoon et al. examined the sensitivity and specificity of ^18^F fluorodeoxyglucose (FDG) PET/MRI in the detection of metastases of rectal cancer and compared them with the current diagnostic standard using pelvic MRI with chest and abdominopelvic CT. They showed a sensitivity of 94% for both methods, whereas specificity was at 73% for the current diagnostic standard and at 98% for ^18^F FDG PET/MRI [6].

Regarding the consequences of misdiagnosis of rectal cancer metastases, cost-effectiveness is a critical factor for deliberating adequate diagnostic instruments. Despite the fact that cost-effectiveness is of increasing importance in the healthcare sector, no study has been published so far that investigated differences between ^18^F FDG PET/MRI and the clinical standard-of-care imaging (SCI) (pelvic MRI with chest and abdominopelvic CT) for initial staging of rectal cancer and its therapeutic consequences from an economic point of view.

The aim of this study is to determine the cost-effectiveness of ^18^F FDG PET/MRI at initial staging of rectal cancer as compared with that of SCI using pelvic MRI with chest and abdominopelvic CT.

## Material and methods

### Model structure

**Fig. 1:**
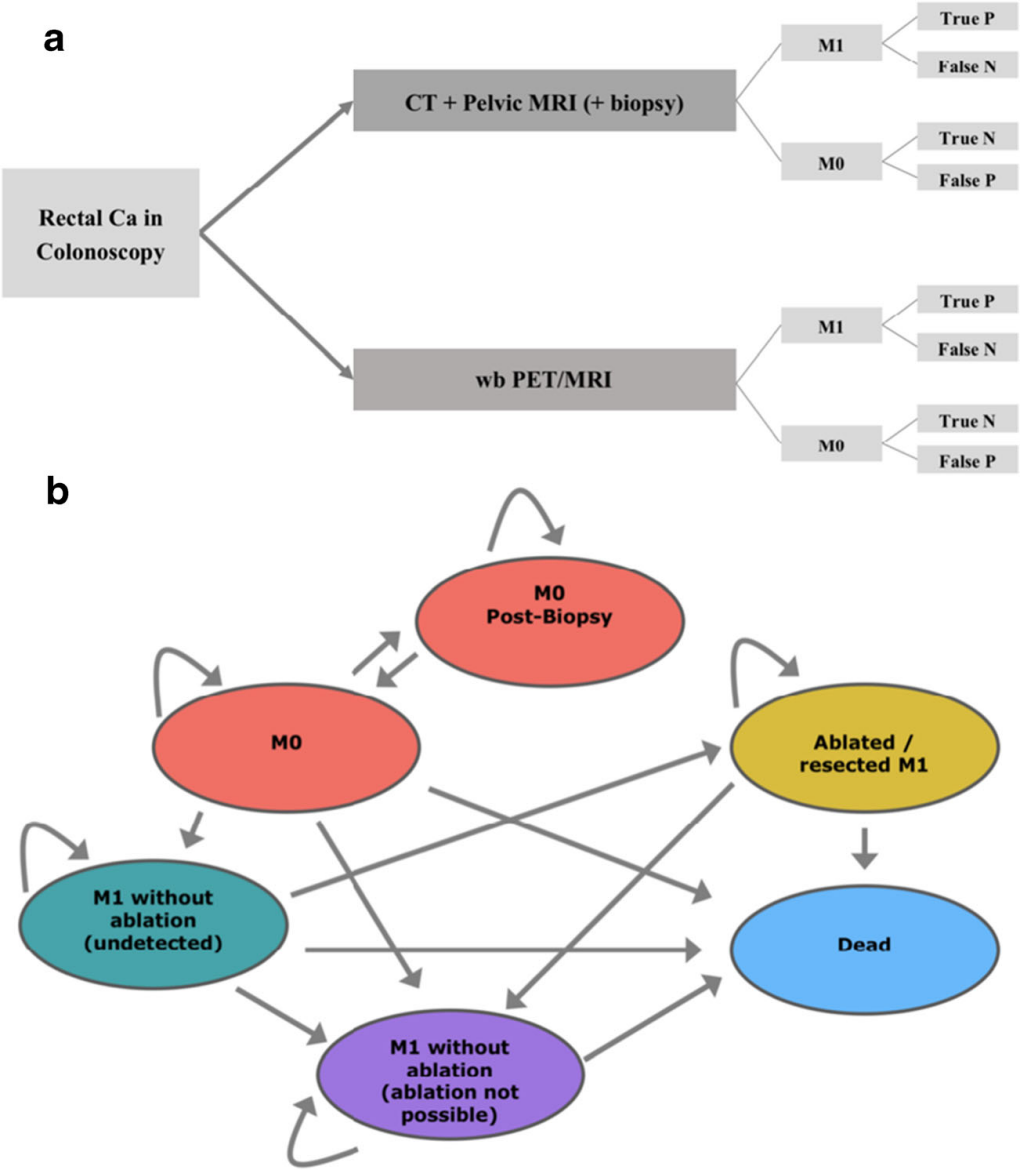
Decision model for strategies CT + pelvic MRI and wb ^18^F FDG PET/MRI. For each outcome, a Markov model analysis was performed (**a**). Markov model with potential states of disease. The first state was determined depending on the outcomes in the decision model (**b**). Ca carcinoma, CT computed tomography, MRI magnetic resonance imaging, PET positron emission tomography, wb whole body, M1 with metastases, M0 without metastases, M Markov model, N negative, P positive

A decision model which included the diagnostic modalities ^18^F FDG PET/MRI with a hepatocyte-specific contrast agent and pelvic MRI + chest and abdominopelvic CT (standard procedure) was created as a decision tree. The model is shown in Fig. 1a. For further evaluation, dedicated decision analysis software (TreeAge Pro version 19.1.1, Williamstown, MA, USA) was used. A Markov transition state model including the following states was applied for analysis of outcome and is shown in Fig. 1b: alive without metastases, alive with undetected metastases, alive with detected metastases not suitable for ablation/resection, alive with ablated/resected metastases, and dead.

### Input parameters

Definition of the model input parameters was performed by review of recent literature (Table 1). The age-specific risk of death was derived from the United States (US) Life Tables [11].

**Table 1:** Model input parameters

Variable	Estimate	Source
Pre-test probability of initial M1 tumor	22%	Noone et al. 2018
Expected age at diagnostic procedure	67 years	Noone et al. 2018
Assumed willingness to pay per QALY	$100,000.00	Assumption
Discount rate	3%	Assumption
Markov model time horizon	5 years	Assumption
Diagnostic test performances		
MRI sensitivity for M1	94 [69.8; 99.8]	Yoon et al. 2019
MRI specificity for M1	98 [90.3; 99.9]	Yoon et al. 2019
CT + pelvic MRI sensitivity for M1	94 [69.8; 99.8]	Yoon et al. 2019
CT + pelvic MRI specificity for M1	73 [59.0; 83.9]	Yoon et al. 2019
Costs (acute)		
PET/MRI	$1443.00	Medicare (Ref. No. 78813)
CT whole body	$586.00	Medicare (Ref. No. 71260 + 74177)
Pelvic MRI	$385.00	Medicare (Ref. No. 72197)
Biopsy	$1375.00	Medicare (Ref. No. 47000)
Probability of biopsy	20%	Expert opinion
Ablation	$4595.00	Medicare (Ref. No. 47382)
Costs (long term)		
M0 yearly	$22,571.80 (first year) $1172.00 (following years)	Joranger et al. 2018
Therapy for patients with M1	$51,706.8	Joranger et al. 2018
Death	0	
Utilities		
M0 yearly	0.79 (first year), 0.87 (following years)	Calderon et al. 2019, Ratjen et al. 2018
M1 without ablation yearly	0.66	Joranger et al. 2018, Fiori et al. 2019
M1 with ablation yearly	0.715 (first year), 0.87 (following years)	Calderon et al. 2019, Helou et al. 2019
QOL after biopsy	0.995	Adapted from Feldmann et al. 2018
Death	0	Assumption
Transition probabilities		
Probability of secondary occurrence of M1 after resection of primarius	3.30%	Augestad et al. 2015
Probability of occurrence of M1 after ablation	29.00%	Lintoiu-Ursut et al. 2015
Probability of death with M0	6.60%	Arias et al. 2019
Probability of death with ablated M1	6.60%	Arias et al. 2019
Probability of death with unablated M1	32.00%	Arias et al. 2019
Percentage of ablatable M1 lesions	17.00%	Brouwer et al. 2018
Percentage of new ablatable lesions in M0	0.56%	Brouwer et al. 2018, Augestad et al. 2015
Percentage of new unablatable lesions in M0	2.74%	Brouwer et al. 2018, Augestad et al. 2016

*QALY* quality-adjusted life years, *QOL* quality of life, *MR*I magnetic resonance imaging, *PET* positron emission tomography, *CT* computed tomography, *M0* no metastases, *M1* with metastases

#### Diagnostic test performances

Sensitivity and specificity values for detection of metastases by ^18^F FDG PET/MRI with a hepatocyte-specific contrast agent and SCI were derived from the literature [6].

#### Costs and utilities

Starting from the US healthcare perspective, costs were estimated based on Medicare data and available literature (Table 1) [6, 9]. Costs for biopsy were added with a factor of 0.2 to all cases of SCI, as according to expert opinions, approximately 20% of cases require further diagnostics via biopsy. Based on literature and conservative assumption, metastases are assumed to be ablatable in 17% of the cases [12].

Annual costs for patients with M0 cancer are derived from follow-up examinations [13]. Initially diagnosed M0 disease and ablatable M1 disease are assumed to result in local resection of the primary tumor.

Utility is measured in the additional quality-adjusted life years (QALY) which are gained through each diagnostic procedure. According to previous literature, the quality of life (QOL) for patients with localized disease was set to 0.83, as therapy and possible complications lead to a reduction of QOL [14–16]. In accordance with the literature, the QOL of patients with metastatic disease was set to 0.66, and QOL after biopsy was set to 0.995 for 1 month [13, 17, 18]. These values were then used for calculations in a Markov model specifically designed as mentioned above. Calculated QOL values in base-case analysis were rounded to three digits to improve readability.

#### Transition probabilities

Transition probabilities were derived from a systematic review of the recent literature and are shown in Table 1. The probability of secondary occurrence of metastases after resection of the primary tumor was assumed to be 3.3%, whereas the probability of occurrence of metastases after ablation (refers to ablation and/or resection) of metastases was assumed to be 29% [19, 20]. The risk of death after successful ablation of metastases was assumed to be similar to the risk without metastases [21]. The age-dependent risk of death was adopted from the US Life Tables endorsed by the Centers for Disease Control and Prevention, National Center for Health Statistics, and National Vital Statistics System [11]. A Similar risk of death by other causes was assumed between patients with and without rectal cancer, as comorbidities decreasing life expectancy seemed unlikely.

### Cost-effectiveness analysis

The pre-test probability of initial M1 malignancy was derived from recent literature [1]. According to current recommendations, a discount rate of 3.0% was assumed [22]. A total time horizon of 5 years after initial diagnosis of rectal cancer was applied for the cost-effectiveness analysis. Willingness to pay (WTP) was set to $100,000 per QALY [23].

For indicating the patients’ state and allowing the evaluation of the modeled outcomes in the Markov model, survival diagrams were created.

A deterministic sensitivity analysis of the costs was performed for determination of the influence of each variable on the model and was visualized as a tornado diagram.

Monte Carlo modeling was used for probabilistic sensitivity analysis. A total of 30,000 iterations were used for calculation of the model.

## Results

### Cost-effectiveness analysis

In base-case analysis with WTP of $100,000 per QALY and a 5-year time frame, SCI resulted in total costs of $51,672 whereas whole-body PET/MRI resulted in total costs of $52,185. Whole-body ^18^F FDG PET/MRI showed an expected effectiveness of 3.542 QALYs versus 3.535 QALYs for SCI. This resulted in an incremental cost-effectiveness ratio of $70,291 per QALY for PET/MRI.

Therefore, from an economic point of view, initial diagnostic management of rectal carcinoma with whole-body ^18^F FDG PET/MRI was slightly more expensive but showed a higher effectiveness compared with SCI in the base-case scenario and a dominance over SCI.

### Markov model

**Fig. 2:**
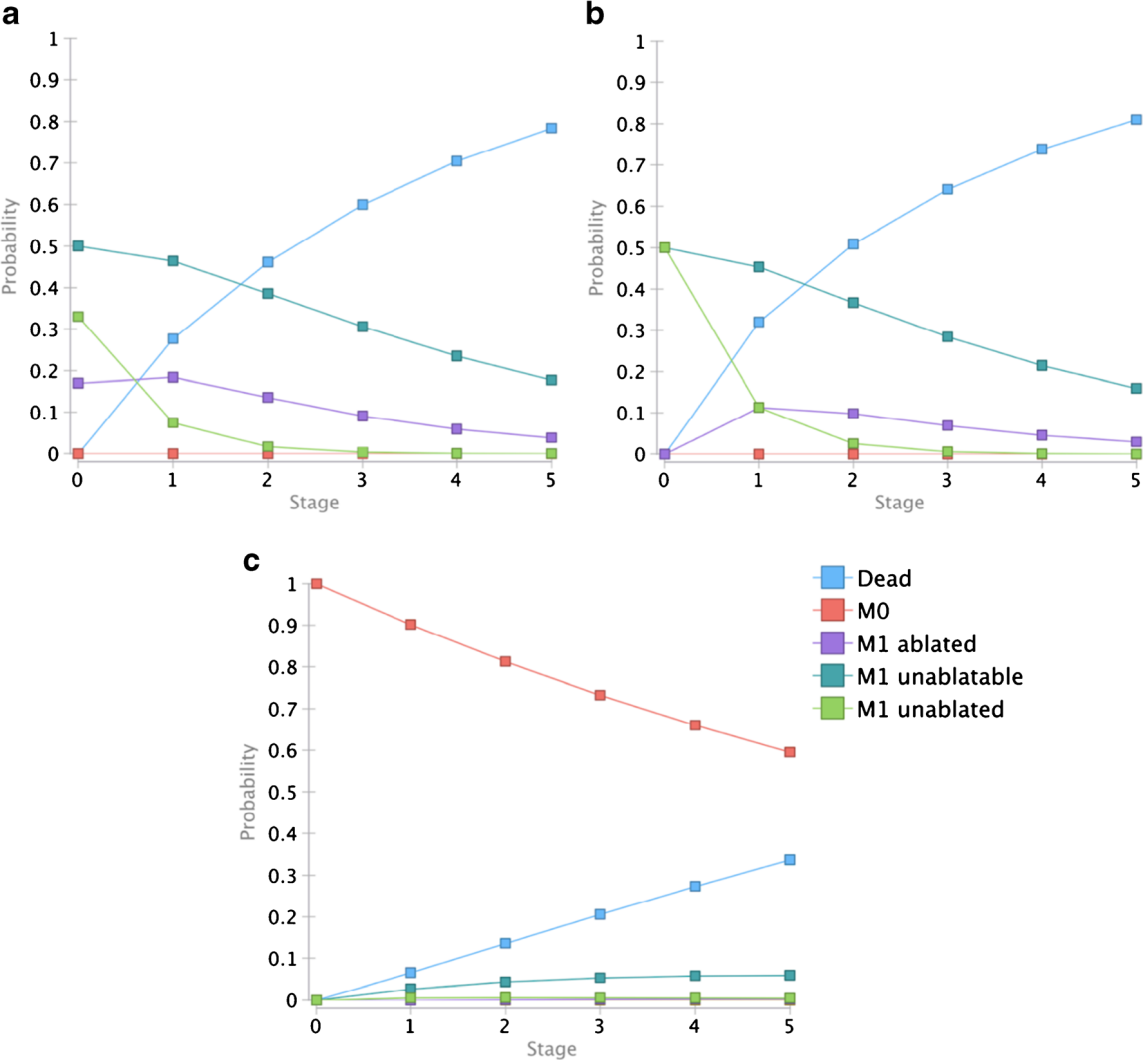
Markov simulation for 5 years. Outcomes for patients with metastatic disease receiving a timely treatment (true positive) (A). Outcomes for patients with metastatic disease receiving a delayed treatment (false negative) (B). Outcomes for patients without metastatic disease (true negative and false positive) (C)

Input parameters of the Markov model lead to the respective state probabilities shown in Fig. 2.

### Deterministic sensitivity analysis

To account for the possibly differing costs of SCI and especially ^18^F FDG PET/MRI with a hepatocyte-specific contrast agent which in combination is not yet established in standard clinical use, a deterministic sensitivity analysis was performed.

**Fig. 3:**
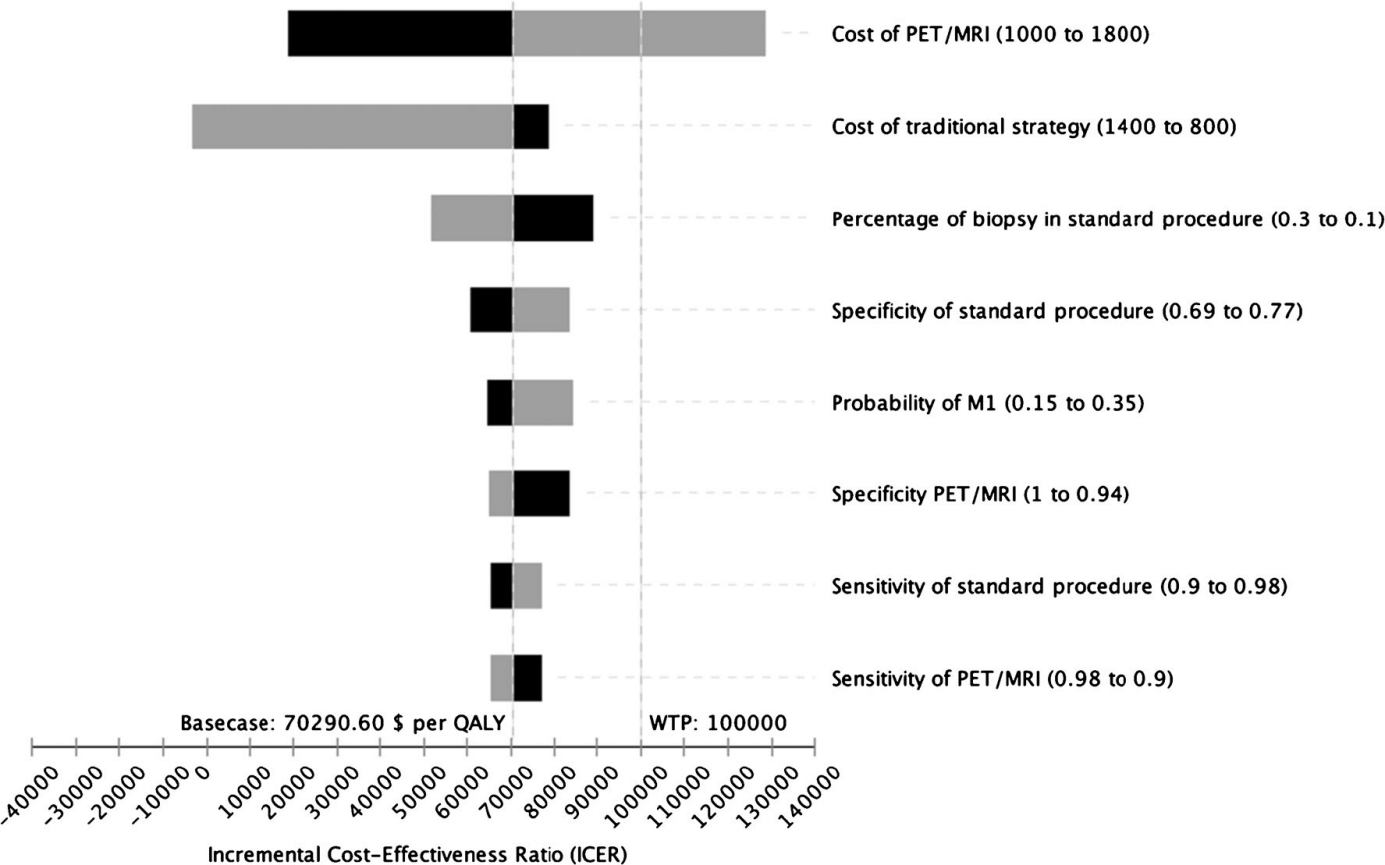
Tornado diagram showing the impact of input parameters on the incremental cost-effectiveness ratio (ICER) starting from the expected value in the base-case scenario. Assuming a willingness-to-pay threshold of $100,000 per QALY, PET/MRI loses its dominance at costs of $1592. For all other parameters investigated, the ICER of ^18^F FDG PET/MRI remained below the willingness-to-pay threshold, indicating the cost-effectiveness of ^18^F FDG PET/MRI in this setting. MRI magnetic resonance imaging, PET positron emission tomography, M0 no metastases, M1 with metastases, QALY quality-adjusted life years

A wide range of $1000 to $1800 was applied for ^18^F FDG PET/MRI and range of $800 to $1400 was applied for SCI. Assuming a WTP of $100,000 per QALY, ^18^F FDG PET/MRI loses its dominance at costs of $1592. For all other parameters investigated, the incremental cost-effectiveness ratio (ICER) of ^18^F FDG PET/MRI remained below the WTP threshold, indicating the cost-effectiveness of ^18^F FDG PET/MRI in this setting as shown in Fig. 3.

### Probabilistic sensitivity analysis

**Fig. 4:**
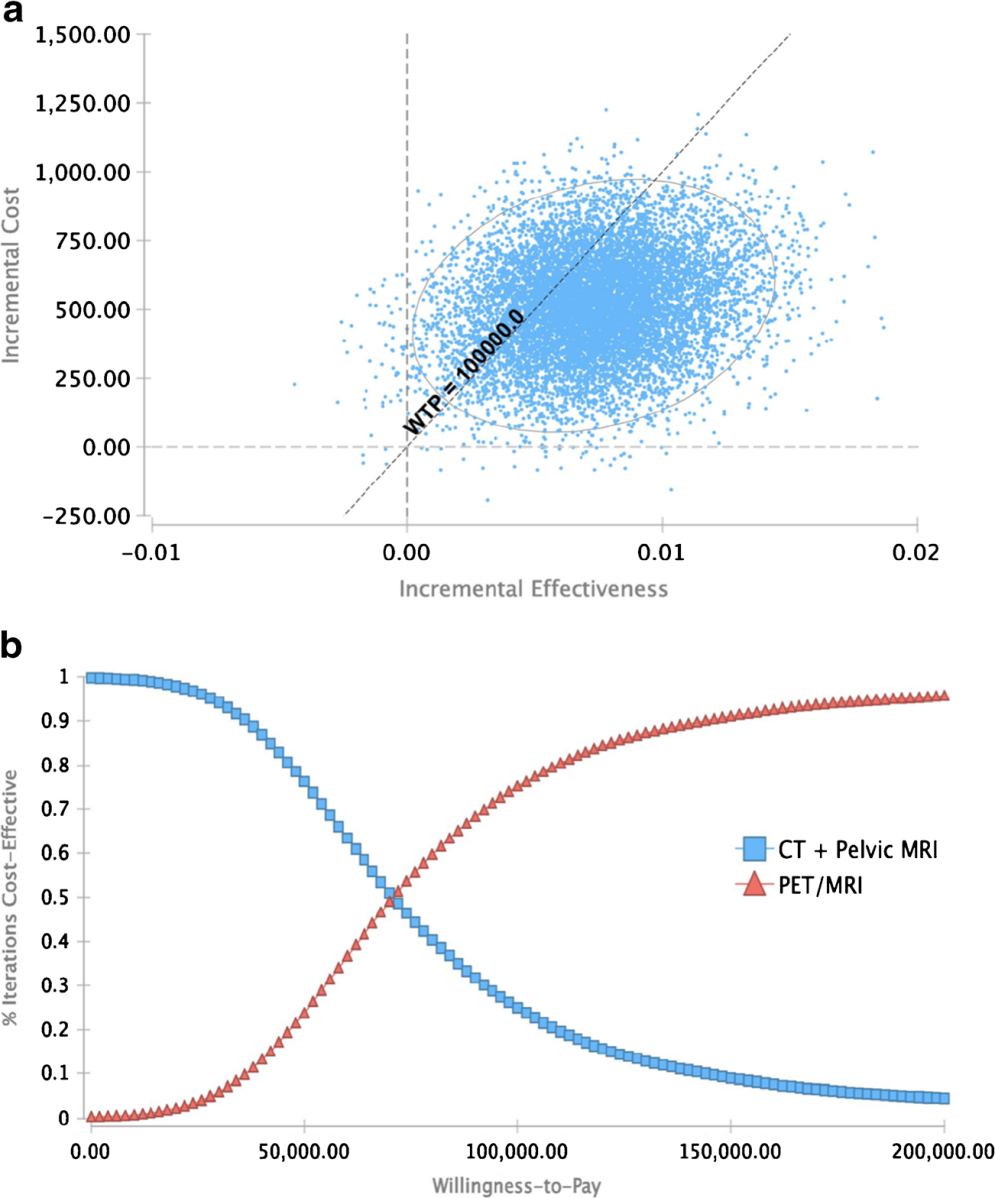
Probabilistic sensitivity analysis utilizing Monte Carlo simulations with 30,000 iterations. Incremental cost-effectiveness scatterplot PET/MRI versus CT + pelvic MRI (**a**). Cost-effectiveness acceptability curve dependent on willingness to pay (WTP) (**b**). PET/MRI is cost-effective in the majority of iterations above a WTP threshold of $70,291

For evaluation of the robustness of the model, a probabilistic sensitivity analysis was performed applying distributions described in Table 1. Results are shown in Fig. 4.

Above a WTP threshold of $70,291, ^18^F FDG PET/MRI is the cost-effective alternative in the majority of iterations.

At a WTP of $100,000 per QALY, ^18^F FDG PET/MRI was cost-effective in 75.7% of iterations. When increasing the WTP, the percentage of iterations being cost-effective for ^18^F FDG PET/MRI also showed an increase, resulting in cost-effectiveness for ^18^F FDG PET/MRI in 95.7% of iterations at a WTP of $200,000 per QALY.

## Discussion

This study demonstrates that ^18^F FDG PET/MRI with a hepatocyte-specific contrast agent is a cost-effective alternative over SCI using pelvic MRI + chest and abdominopelvic CT with fine-needle biopsy for inconclusive cases in initial diagnosis of rectal cancer. Fine-needle biopsy is not uncommon in patients with colorectal cancer since the liver is one of the main sites of colorectal cancer metastases [19, 24]. Therefore, the inclusion of fine-needle biopsy in the Markov model of this study was an important factor on costs, quality of life, and effectiveness.

In a study by Sivesgaard et al., MRI performed significantly better than both contrast-enhanced CT and combined ^18^F FDG-PET/CT for detection of hepatic metastases of rectal cancer [25]. Furthermore, the high sensitivity and specificity of PET/MRI for determination of the M state in malignant diseases as compared with those of other diagnostics had been proven throughout the last years [8, 26]. A study by Queiroz et al. showed the high accuracy of PET/MRI in detection of metastatic disease in initial staging of rectal cancer, whereas sensitivity and specificity were even higher in a study by Yoon et al. using a hepatocyte-specific contrast agent [6, 27].

This study outlines the economic advantages of PET/MRI over SCI focusing on initial M staging of rectal cancer. Additionally, a study recently published by Catalano et al. pointed out the advantages of PET/MRI over SCI in staging of N status, further underlining the cost-effectiveness of PET/MRI over that of SCI in this scenario [28].

Mayerhoefer et al. were the first to make initial economic approaches in the use of PET/MRI in oncologic diagnostics in a variety of diseases [9]. In contrast to Mayerhoefer et al., who gave an overview on application of PET/MRI in several indications, this study demonstrates cost-effectiveness in a clearly defined clinical scenario.

We derived costs of ^18^F FDG PET/MRI from Medicare data. Nevertheless, in recent literature, costs of PET/MRI are assumed to be lower when used in daily clinical practice [9]. Our study shows that ^18^F FDG PET/MRI is cost-effective at assumed costs of $1443 but loses its dominance at costs of $1592 for ^18^F FDG PET/MRI, indicating the relevance of this factor in our model.

Further deterministic sensitivity analysis showed good reliability of the results regarding other input parameters, variation of costs, sensitivity, and specificity of SCI and ^18^F FDG PET/MRI, as well as the probability of occurrence of metastases in a wide range still leads to the cost-effectiveness of ^18^F FDG PET/MRI over that of SCI. Additionally, the sensitivity and specificity of MRI in this study are based on examinations using a hepatocyte-specific contrast agent, resulting in higher sensitivity for MRI regarding hepatic metastases, which are common in rectal cancer [6].

Regarding limitations, our Markov model does not differ between local tumor states as this would outrange the scope of this study. Future studies could investigate the cost-effectiveness of ^18^F FDG PET/MRI in relation to local tumor extent as the probability of M1 depends on the T state.

Moreover, our study only includes SCI and whole-body ^18^F FDG PET/MRI and does not take into account other means for initial diagnosis of rectal cancer, such as ^18^F FDG PET/CT or CT only [26, 29]. Those were outperformed by MRI in former studies; nevertheless, its cost-effectiveness compared with that of PET/MRI in rectal cancers is yet to be determined [28].

Furthermore, our Markov model does not allow patients with ablated / resected metastases to enter the state of undetected metastases as this case seems very rare and input values would be unlikely to be available in literature.

In our study, we assumed biopsy to be performed in approximately two-thirds of unclear cases in SCI. Nevertheless, in clinical practice, this number may vary and follow-up examinations or other diagnostic means may be used [25]. Additionally, fine-needle biopsy is an invasive means and can cause needle-tract tumor seeding, with possible complications especially in cases eligible for ablation of metastases [30].

European and American guidelines both agree that ^18^F FDG PET/CT has no relevance in the diagnostic workup of newly diagnosed CRC but state it can be performed in patients with resectable liver metastases of CRC to avoid an unnecessary laparotomy or in equivocal CT findings, whereas ^18^F FDG PET/MRI is not mentioned at all [31].

Especially as ^18^F FDG PET/MRI with a hepatocyte-specific contrast agent is a one-stop solution and a non-invasive diagnostic modality with high diagnostic accuracy, results of this study support the potential of ^18^F FDG PET/MRI for future use in the initial staging of newly diagnosed rectal cancer. Nevertheless, the choice of diagnostic modalities depends on other factors such as availability of ^18^F FDG PET/MRI, already performed diagnostic workup, or local tumor extent.

In conclusion, this study demonstrates possible advantages of ^18^F FDG PET/MRI over SCI of initial diagnosis of rectal cancer in a primary economic approach, showing high robustness to variability of input data. Nevertheless, results are based on initial approaches and confirmation is the subject of further studies which might include complementary diagnostic modalities and examine the influence of local tumor spread on cost-effectiveness.

## Data Availability

All data is presented within the manuscript.
